# Understanding the impacts of chronic pain on autistic adolescents and effective pain management: a reflexive thematic analysis adolescent–maternal dyadic study

**DOI:** 10.1093/jpepsy/jsae004

**Published:** 2024-02-07

**Authors:** Abbie Jordan, Amelia Parchment, Jeremy Gauntlett-Gilbert, Abigail Jones, Bethany Donaghy, Elaine Wainwright, Hannah Connell, Joseline Walden, David J Moore

**Affiliations:** Department of Psychology, University of Bath, Bath, United Kingdom; Centre for Pain Research, University of Bath, Bath, United Kingdom; NIHR Applied Research Collaboration Greater Manchester, University of Manchester, Manchester, United Kingdom; Bath Centre for Pain Services, Royal United Hospitals Bath, Bath, United Kingdom; Centre for Health and Clinical Research, University of the West of England, Bristol, United Kingdom; Department of Psychology, University of Bath, Bath, United Kingdom; Centre for Pain Research, University of Bath, Bath, United Kingdom; Research Centre for Brain & Behaviour, Liverpool John Moores University, Liverpool, United Kingdom; Centre for Pain Research, University of Bath, Bath, United Kingdom; Aberdeen Centre for Arthritis and Musculoskeletal Health (Epidemiology Group), School of Medicine, Medical Sciences and Nutrition, University of Aberdeen, Aberdeen, United Kingdom; Bath Centre for Pain Services, Royal United Hospitals Bath, Bath, United Kingdom; Department of Psychology, University of Bath, Bath, United Kingdom; Research Centre for Brain & Behaviour, Liverpool John Moores University, Liverpool, United Kingdom

**Keywords:** pain, autism, adolescents, parents, qualitative

## Abstract

**Objective:**

Sensory elements are core features in chronic pain and autism, yet knowledge of the pain experience in autistic adolescents is limited. Little is known regarding how autistic adolescents experience chronic pain, manage their pain and perceive psychological treatment for their chronic pain.

**Methods:**

Ten autistic adolescents (6 female, 3 male, and 1 self-identified as agender) with chronic pain and their mothers (*n* = 10) participated in semistructured interviews concerning their perceptions of living with chronic pain. Participants were recruited from U.K. pain management services. According to preference, interviews were conducted individually (*n* = 10) or dyadically (*n* = 10 participants across 5 dyads). Data were analyzed using inductive reflexive thematic analysis.

**Results:**

Two themes were generated. Theme 1, “overstimulated and striving for control” described how adolescents’ experience of heightened sensitivity enhanced adolescents’ levels of anxiety and subsequent pain, illustrating a reciprocal relationship between anxiety, pain, and sensory elements. Theme 2, “not everyone fits the mold” captured how autistic adolescents positioned themselves as distinct from others due to the unique nature of being autistic and living with pain. This sense of difference negatively impacted adolescents’ ability to engage with and benefit from the standard treatment for chronic pain.

**Conclusions:**

Findings suggest that autistic adolescents living with pain experience pain and face barriers to effective pain treatment. Our results identify the need for educational resources to facilitate clinicians to better understand the experience of autistic adolescents living with pain. In turn, such understanding may improve treatment and outcomes in this population.

Adolescence is a developmental period characterized by multiple key tasks that individuals complete as they transition into adulthood. Such tasks may be particularly challenging for adolescents who live with physical health conditions including chronic pain. Recent worldwide data identified that chronic pain is common, with Gobina and colleagues (2019) reporting that 44.2% of 11- to 15-year-olds experience chronic pain. For a sizable minority, the experience of chronic pain is associated with deleterious adolescent physical, social, emotional, scholastic, and developmental functioning (Jones et al., 2020; Murray et al., 2020).

An understanding of pain experience in autistic adolescents remains limited (Moore, 2014), with both pain and autism involving an overriding sensory element (American Psychiatric Association, 2013; Raja et al., 2020). Autistic individuals have been suggested to have similar or lower thresholds for pain compared with their neurotypical peers (Vaughan et al., 2020); however, autistic adults do show significantly greater levels of pain-related fear that predicts greater ratings of pain presented at suprathreshold levels (Failla et al. 2020). Evidence shows that 14% of children and adolescents within a tertiary pain setting displayed clinically significant autistic traits, greater than the 1%–2% population prevalence, with a higher prevalence of autistic traits in girls than in boys (Lipsker et al., 2018). Notably, Whitney and Shapiro (2019) identified higher rates of chronic pain in autistic 6 to 16-year-olds (15.6%) compared with neurotypical 6- to 16-year-olds (8.2%).

Experiencing chronic pain typically involves seeking access to healthcare services, yet there are widespread reports that autistic individuals experience a range of inequalities regarding their healthcare experiences. Autistic individuals have shorter life expectancies and poorer health than their nonautistic peers and report receiving lower-quality healthcare (Smith DaWalt et al., 2019). Research has revealed that barriers faced by autistic individuals in accessing healthcare include challenges with health anxiety, communication under stress, and a lack of autism awareness in staff (Hampton et al., 2022). Autistic populations are also at greater risk of contact with health services due to co-occurring mental and physical health conditions (Donaghy et al., 2022; Hanlon et al., 2022).

Consideration of contact specifically with services for chronic pain within this population is however lacking, interventions are often psychologically focused, typically comprising cognitive-behavioral therapy (CBT) or acceptance and commitment therapy (ACT) (Sturgeon, 2014). These interventions aim to minimize distress and negative pain outcomes by changing unhelpful pain cognitions and behaviors (Kaiser et al., 2015) or accepting pain distress openly while still living a meaningful life (ACT) (Kemani et al., 2018). Although such interventions seemingly provide improvements in pain intensity and pain-related functioning for some adolescents, (Gauntlett-Gilbert et al., 2013), evidence has not yet shown whether these interventions are effective for all adolescents (Fisher et al., 2019). One factor that influences how individuals experience psychological therapies differently and increase the likelihood of chronic pain is an autism diagnosis (Jones & Shivamurthy, 2022).

Evidence suggests that CBT may be effective for decreasing symptomatology and severity of mental health conditions for autistic children and young people (Linden et al., 2023). However, CBT relies on higher levels of psychological flexibility, which have been shown to differ between autistic and nonautistic adolescents, suggesting a need to adapt CBT to meet individual needs (Rotheram-Fuller & MacMullen, 2011). Similarly, some evidence exists concerning the use of ACT for pediatric chronic pain in neurodivergent populations (Balter et al., 2021). While promising, such research does not provide any understanding of how autistic adolescents understand and potentially benefit from any such psychologically led treatment.

To address this knowledge gap, the present study sought to understand how autistic adolescents living with chronic pain and their parents experience chronic pain, manage their pain, and perceive psychological treatment for their chronic pain. A qualitative design was undertaken to enable authors to focus on generating an in-depth understanding of the experiences and perceptions of autistic adolescents and their parents. A reflexive thematic analytical approach was adopted to facilitate the generation of patterns across the data set rather than focusing on cases at a more individual level (Braun & Clarke, 2021).

## Methods

### Design

This study employed an exploratory dyadic qualitative research design with the purpose of developing a rich, in-depth understanding of how autistic adolescents and their parents experience pain, manage pain and perceive psychological treatment of chronic pain.

### Participants

Following the University of Bath (ref 20-205) and U.K. National Health Service ethical approvals (IRAS 279953), a total of 10 parents and 10 adolescents were recruited into the study. Adolescents were eligible to take part in the study if they were aged 11–20 years, experienced pain for a duration of 3 months or longer (chronic pain), provided informed consent/assent, and had been referred to a specialist tertiary pain service in the South-west of England or received treatment at that service within the past 5 years. Typical interdisciplinary therapeutic services offered by this tertiary-level pain service (e.g. 3-week residential programs) are based on ACT and have been described in detail elsewhere (Kemani et al., 2018). Additionally, all adolescent participants were required to meet at least one of the following criteria: (1) have a pre-existing diagnosis of an autism spectrum disorder, (2) have written, formal clinical team recognition of autism spectrum disorder traits after inpatient assessment, and (3) score above clinical cutoff on a parent-rated screening instrument for the detection of an autism spectrum disorder (Social and Communication Disorders Checklist, Skuse et al., 2005). Critically all participants identified as autistic; given that late diagnosis is common, especially among autistic women and those with more typical verbal communication (Bargiela et al., 2016), this decision to recruit self-identified autistic people was made purposively to ensure that the breadth of likely autistic experiences of chronic pain and pain management was represented. Participants were recruited in dyads, with an accompanying parent/carer being eligible to participate in the study if they were considered the primary parent/carer of an adolescent (11–20 years) who met the above eligibility criteria. Exclusion criteria included being unable to read, write, or speak English to a level required to participate in the interview. Details of participants can be found in Table 1. Adolescents were aged 15–19 years (mean age 16.50 years, *SD* 1.51 years), with adolescents reporting a mean pain duration of 161.67 months (*SD* 105.34 months, median 96 months) and a mean current pain intensity of 6.9 (*SD* 2.02, median 8.0) using a 10-point numerical rating scale (no pain—worst pain ever). Six adolescents identified as female, three as male, and one as agender (participant’s choice of term). Maternal age ranged from 37 to 55 years (mean age 46.3 years, *SD* 5.44 years). While all parents were invited to take part in the study, all participating parents were mothers and are subsequently referred to as a maternal sample from hereon. All 20 participants identified as being of white ethnicity.

**Table 1.** Demographic characteristics of adolescent/mother dyads.

Dyad number and nature of interview	Adolescent pseudonym	Adolescent age (years)	Adolescent gender	Adolescent pain duration, months	Maternal age, years	**Adolescent self-reported diagnosis/diagnoses** ^a^
1 (individual)	Emily (A)	18	Female	17	55	Complex regional pain syndrome with dystonia
2 (individual)	Jack (A)	15	Male	120	37	Hypermobility spectrum disorder
3 (individual)	William (A)	15	Male	240	47	Chronic widespread pain syndrome
4 (dyadic)	Isabella (A)	15	Female	360	45	Myalgic encephalomyelitis/chronic fatigue syndrome
5 (dyadic)	Jamie (A)	17	Agender	96	41	Chronic widespread pain syndrome, Charcot-Marie-Tooth disease
6 (individual)	Jacob (A)	15	Male	—^b^	52	Chronic widespread pain syndrome
7 (dyadic)	Chloe (A)	18	Female	70	50	Complex regional pain syndrome
8 (dyadic)	Olivia (A)	16	Female	120	49	Ehlers–Danlos syndrome, complex regional pain syndrome, postural orthostatic tachycardia syndrome, symphysis pubis dysfunction, sacroiliac joint dysfunction, irritable bowel syndrome, dysmenorrhea
9 (dyadic)	Amy (A)	17	Female	204	42	Ehlers–Danlos syndrome, postural tachycardia syndrome, gastroparesis, eosinophilic gastrointestinal disease, obsessive compulsive disorder, anxiety, depression, hyperthyroidism
10 (individual)	Sophia (A)	19	Female	228	45	Complex regional pain syndrome, hypermobility syndrome

a Adolescents’ self-report of pain-related diagnoses provided by healthcare professionals.

b Missing data.

Sample size for this study was guided by distinct study characteristics, including the study objectives, the qualitative expertise of the researchers, and the quality and richness of the data (Malterud et al., 2016). Subsequently, an approximate sample size of 16–26 participants (8–13 dyads) was considered to be appropriate due to the perceived richness of the data, the specific focus of the study aims, and the authors’ extensive proficiency in employing qualitative methods including inductive reflexive thematic analysis, as adopted in this study. Our sample of 20 participants (10 dyads) met these sample size parameters. Sample size in this study is congruent with other studies that have used reflexive thematic analysis to examine interview data (e.g., Jordan et al., 2023).

### Procedure

Participants were recruited through specialist tertiary pediatric pain services in the South-west of England and were approached in one of three ways: (a) by a clinician during their initial assessment appointment, (b) by a clinician during their residential treatment program, or (c) via email sent to past patients identified from a search of clinical records. Eligible participants received an email inviting them to take part in the study along with an information sheet. Eighteen dyads received further information about the study, with one dyad expressing that they did not wish to participate. Seventeen dyads were sent age-appropriate links to a short online survey with consent statements and brief demographic questions. All mothers were required to provide informed consent for their own study participation as well as for their adolescents (if aged 11–15 years). Adolescents aged 11–15 years were asked to provide informed assent while adolescents aged 16–20 were required to provide informed consent. Seven dyads did not complete the consent forms, with a total of 10 dyads participating in the study.

Participants were given the choice to be interviewed together as a dyad, or individually. A total of 5 dyadic interviews (10 participants) were conducted with the remaining 10 participants opting for independent interviews (5 mothers, 5 adolescents). Individual adolescent and maternal interviews were scheduled closely together, with the initial interview comprising whichever participant was first available to participate. Recruitment occurred between November 2020 and September 2021.

### Interview schedule

The interview schedule was created collaboratively with input from (1) academic colleagues with experience in pain and autism, (2) clinical colleagues with relevant experience of working with autistic adolescents who experience pain and their parents in addition to (3) an autistic adolescent working in the area of chronic pain. The research team initially suggested potential topics for the interview schedule based on their professional or lived experience. Topics were reviewed and subsequently developed into open-ended questions, which sought to elicit detailed responses from participants about their own or the autistic adolescent’s experience of living with chronic pain. Interview questions were not directly informed by any specific relevant theories. Prompts and probes were included to enable detailed discussion of participant responses. A draft interview schedule was pilot tested by an autistic adolescent and amended based on their feedback. Interview schedules were identical for adolescent and maternal participants with the exception of mothers being asked about their adolescent’s experience rather than adolescents being asked to self-report on their own experience. Questions focused on challenges that autistic adolescents with pain may face (e.g., rigidity of thinking) and how this may impact their experience of pain, its management, and experiences of pain treatment. The same interview schedule was used throughout for adolescents and mothers although conversations were guided by the participant, enabling the interviewer to tailor the guide to each specific participant (Morgan Brett & Wheeler, 2022).

The adolescent interview schedule can be found in the supplementary material. Interviews were conducted via video conferencing software, with interviews ranging from 18 to 83 min (mean duration, 43 min). Specifically, the mean duration for interviews was as follows: young person (36 min), mother (47 min), and dyadic (48 min). Approximately, 50% of dyadic interviews were devoted to maternal responses and 50% to the young person. Interviews were transcribed verbatim, with identifiable information removed to preserve anonymity. On study completion, all participants received a £20 shopping e-voucher and were debriefed. The data underlying this article cannot be shared publicly due to ethical permissions.

### Data analysis

Inductive reflexive thematic analysis was employed to explore and interpret patterns of meaning within the interview data. Reflexive thematic analysis is an established qualitative interpretatively focused approach that is commonly used to analyze qualitative data (Thompson et al., 2022). Unlike other qualitative approaches such as interpretative phenomenological analysis (Smith, 1996), reflexive thematic analysis is theoretically flexible, meaning that it can be used with a diverse range of theoretical frameworks (Braun & Clarke, 2022).

We embraced a critical realist perspective for our analyses. Reflexive thematic analysis has a longstanding tradition of employing a critical realist framework for examining qualitative data (Braun & Clarke, 2022). In this study, the position of critical realism posits that interview data brought a specific interpreted reality that was firmly situated in a particular situation (e.g., autistic participants having been recruited from a national pain setting), with authors subsequently interpreting this context-specific reality through the use of reflexive thematic analysis (Braun & Clarke, 2022).

Data analyses followed the six-stage approach described by Braun and Clarke (2022), which comprises (1) familiarizing oneself with the data through immersion in the anonymized interview transcripts; (2) generating initial codes in the interview data to reflect potentially interesting data of relevance to the research question; (3) generating initial themes to elucidate patterns of shared meaning across the interview data; (4) developing and reviewing themes to consider how well the initial themes fit the data and how well the analyses “work”; (5) refining, defining, and naming themes to ensure that individual themes are “stand-alone”; and built around a single defining concept; (6) writing up findings that involves an iterative writing and rewriting process.

The dataset was used as a starting point for engaging with meaning, adopting a “data-driven” approach to analysis (Braun & Clarke, 2022). Codes were organized using NVivo (QSR, 2022) and initial themes were developed. Authors reviewed the initial themes and refined them in an iterative manner until themes were perceived to accurately reflect the meaning found in the data. Next, authors discussed coding and themes, reflecting upon the situated interpretation of data. This enabled the naming of themes and write-up of the analytical account.

### Quality and rigor

To ensure a robust analytical process, the authors considered key aspects of quality and rigor. Credibility, the degree to which the results relate to the area of investigation (Hannes et al., 2011), was achieved through the involvement of autistic individuals throughout the project. Credibility checks were built into the analysis through author discussions as the analysis progressed. Confirmability, how much the results are grounded in the data (Elliott et al., 1999), was attained through the inclusion of quotations across most dyads in the results section. Finally, dependability, the clarity of the research process (Shenton, 2004), was attained through detailing a clear outline of the study design and methods.

Importantly, reflexivity is a critical element of reflexive thematic analysis (Braun & Clarke, 2019) and as authors, we wish to reflect on our role as researchers within the study process. The study team comprised a range of clinicians and researchers with expertise in pain (A.J. [first author] A.F.J., H.C., J.G.G., D.J.M., A.P., E.W., B.D.) and autism (D.J.M., B.D., J.W.). In relation to the participants, the study team is aware that they do not form part of the participant group in terms of being an autistic adolescent or parent of an autistic adolescent living with chronic pain. However, one author is autistic and five authors are themselves parents, some with children of a similar age.

## Results

Living with chronic pain as an autistic adolescent added further complexity to the lives of adolescents beyond that associated with living with pain alone. Two themes were generated during inductive reflexive thematic analysis (Braun & Clarke, 2019), which encapsulate the unique perspectives of both autistic adolescents and their parents in relation to how adolescents perceive, experience, and manage chronic pain. Themes comprised (1) overstimulated and striving for control and (2) not everyone fits the mold. Both themes are presented in turn below with quotations provided as exemplars of our interpretation. Anonymized participant identifiers are included with quotations along with a note of whether the interview was conducted as a dyad (e.g., Chloe, dyad or Chloe’s mother, dyad) or individually (Jack, individual or Jack’s mother, individual).

### Overstimulated and striving for control

Adolescents described their experience of heightened sensitivity to particular sensory triggers and how this enhanced sensitivity related to their levels of anxiety and subsequent pain. Conversely, for some, pain itself was considered as “a trigger for everything” (Isabella’s mother, dyad). While a link between pain intensity and anxiety is commonly seen in pediatric pain settings, the nature of this relationship was intensified for autistic adolescents as a result of exposure to sensory triggers in daily life, such as unexpected sounds and touch. The relationship between anxiety and pain was often reciprocal in nature and frequently triggered by overstimulation of the senses. Olivia highlighted the cascading relationship between sensory stimulation, and their experience of anxiety and pain, as exemplified below.

*Noises can trigger my anxiety a lot as well and lead to panic attacks…the panic attacks kind of make everything worse because my panic attacks can be very severe to the point that I hyperventilated severely and that kind of causes my pain to be worse (Olivia, dyad).*

Jacob further emphasized how the interconnectedness of sensory experiences, pain and anxiety determined the day-to-day fluctuations in their experience of living with chronic pain. A tri-directional relationship between factors was highlighted, whereby the presence of one factor (e.g., anxiety) intensified the presence of another (e.g., pain or sensory sensitivity), and vice versa. Consequently, sometimes, the adolescents experience an unrelenting cycle of heightened pain, anxiety, and sensitivity to sensory triggers.

*If I’m on a high pain day my anxiety is high, I have a lot more reaction to sensory stuff and I get a lot more overwhelmed just by normal…day to day noises and stuff in the house compared to low pain day. (Jacob, dyad)*

Managing this dynamic relationship between pain, anxiety, and sensory stimulation often involved increasing routine and ritualistic behaviors. These behaviors offered a clear mechanism by which the adolescents could shield themselves from unexpected sensory triggers and reduce elevated pain-related anxiety. As Jack highlighted, routine and ritualistic behaviors fulfilled a useful function in terms of providing comfort and a sense of predictability amidst the unpredictable day-to-day experience of chronic pain, which served to relieve the experience of pain itself.

*Ritualistic actually helps…with the pain…because it…either provides me a distraction or something else to do you know things that are associated in my head as comforting or relieving (Jack, individual).*

Conversely, while autistic adolescents typically focused on describing their experience and management of the complex relationship between sensory experience, anxiety, and pain, mothers instead identified a “cutoff” point at which they perceived adolescents to become overwhelmed by these interconnecting factors. When reaching this threshold, mothers described how adolescents became incapacitated and unable to control pain due to an unmanageable sense of sensory overload. In these instances, adolescents withdrew from others who could support them to manage pain as a sense of self-preservation.

*When everything else gets too overwhelming I think pain gets too overwhelming for her and that’s it then… complete shutdown, then she can’t help herself (Chloe’s mother, dyad).*

Once mothers perceived their adolescents to be overwhelmed, they reported a sense of personal accountability for re-engaging their adolescents in the management of their anxiety and pain. To enable this, some mothers encouraged their adolescents to engage in routine and ritualistic behaviors to aid coping. This is highlighted by Jamie’s mother who discussed encouraging her adolescent to engage in routine cleaning to alleviate cognitive distortions experienced during distressing anxiety, pain, and sensory-provoking situations. Most strikingly, the use of the word “we” emphasized a strong sense of shared responsibility experienced by Jamie’s mother and a dyadic approach to engaging her adolescent in pain management in the context of them experiencing sensory overload.

*The way we can control her catastrophizing is to have a routine to control, well it doesn’t really control it…but it’s …her coping isn’t it (Jamie’s mother, dyad).*

Routine was also sought around the management of pain medications. For some adolescents, this meant inflexibly assuming sole responsibility for managing their pain medications, demonstrating a deeper understanding of their mechanisms of action, dosage, and drug interactions compared with their parents. This is illustrated below by Chloe and her mother. Administering medications occurred within rigid timeframes for Chloe, highlighting the prominence of routine and ritualistic behaviors in the management of pain as an autistic individual. In this context, both Chloe and her mother perceived this sense of control and routine as a positive driver for encouraging proactive and beneficial pain management behaviors.

*She’s eighteen now but from the age of what fifteen, sixteen, I have not been in charge of her meds… she’s been in charge of her meds because she knows the exact minute and hour that she’s due to take them when they’re wearing off what she can have what she can combine with what (Chloe’s mother, dyad).*

*Well I’m like all my different medications and stuff I've learnt all about them how they work… and especially the ones that have got multiple different uses (Chloe, dyad).*

For other adolescents, rigidity concerning pain management involved informally allocating responsibility to a single “other” nominated individual, such as a parent, resulting in a continued sense of reliance on this individual for pain relief.

*He won’t let Papa fill up the medication and dosset box because the first person that did it was me. Now it’s my job whether I wanted it to be or not…it’s that because that’s how it was so when things start a certain way, they have to be carried out a certain way…he doesn’t particularly want his dad to do it because he’s always relied on me so with his pain he will stick out the pain until I’m…back from work (Jack’s mother, individual).*

As exemplified above, Jack’s inability to allow his father to provide medication when required was problematic, resulting in untreated pain when the nominated parent was absent. In such instances, this sense of control was deemed to be “unhelpful” in the context of providing appropriate pain relief, yet for other adolescents, this sense of ritual and control facilitated increased autonomy for self-managing pain.

### Not everyone fits the mold

Adolescents in this study positioned themselves as different from their peers, with Emily, contrasting her pain experiences with that of “a relatively normal person” (Emily, individual). As well as identifying this sense of difference themselves, autistic adolescents felt that others also perceived them differently from their peers. An autistic identity as well as chronic pain was perceived to provide a sense of double difference as a result of living with both conditions. This perceived sense of difference was further heightened when acknowledged by others.

*It’s so tiring and so exhausting being in pain and then it’s even more so by having autism and then by having it suddenly pointed out that you are so completely different I think it can be quite isolating (Jack’s mother, individual).*

Adolescents were cognizant of their sense of difference from peers who themselves lived with chronic pain but were not autistic. They felt a lack of “fit” for themselves within the chronic pain community, which subsequently impacted their ability to engage with and benefit from pain treatment. William’s mother exemplifies this below, explaining how this perceived sense of difference extended to treatment settings, making engagement in group-based treatments particularly challenging:

*He [William] wasn’t interested in working with any of the people [adolescents on residential treatment programme], they all set up like a private snapchat to kind of support each other and have fun out of the group and he wasn’t interested at all…As the week progressed we kind of became sort of a little bit more separated from the group…because he really wasn’t participating outside of the group at all, “I’ve got nothing in common with these people, why should I be talking to these people and so therefore I don’t want to talk to these people” (William’s mother, individual).*

Further, perceived differences for autistic adolescents living with chronic pain related to communication about pain, with many communicating pain in atypical ways (e.g., laughing) compared with nonautistic adolescents. Despite the unique nature of pain-related behaviors, over time, mothers learned to identify the ways in which their adolescents expressed pain (e.g., via anger, withdrawal and reduced behavioral reactivity), while clinicians were reported to struggle to identify such behaviors such as laughing to be indicative of the experience of pain.

*When I was a kid and I was in pain I would laugh… it took maybe an extra year or two to actually have it recognised that this isn’t just a small issue this isn’t something I find funny, there’s something worse here (Jack, individual).*

*We had a little bit of a problem at first with…physios or doctors not understanding that when he’s laughing, he’s in pain so we were almost like sort of brushed off for him so I think maybe not being taken seriously or understanding quite the level of pain he was in (Jack’s mother, individual).*

For adolescents, this differing presentation of pain resulted in numerous challenges, reinforcing a sense of the double difference between autistic adolescents with chronic pain and their peers. Additionally, such atypical behaviors resulted in reduced access to effective pain management since clinicians were reported as struggling to identify reduced behavioral reactivity as an indicator of pain in autistic adolescents.

Adolescents described how they felt disadvantaged by a perceived “one-size-fits-all” approach to psychological management of chronic pain, noting difficulties with tasks that involved abstract and metaphorical thinking, and used conceptual scenarios during therapy sessions. Exercises using these approaches aimed to engage attendees with their emotions, thoughts, and feelings in order to observe how these factors might interconnect to impact their experience of pain and its management. However, such exercises were problematic for some autistic adolescents, whose cognitive profile and abilities differed from their nonautistic peers. For example, they struggled to think about things concretely and therefore struggled to engage in abstract or conceptual thinking in relation to their experience of pain.

*Some of the mindfulness ones are like imagine you’re up a mountain and things like that I’m just like “I know I’m not up a mountain”…I was just thinking “I can’t imagine being anywhere other than where I am” (Chloe, dyad)*

Consequently, autistic adolescents could not experience the benefits of these exercises for the management of their pain and associated functioning, further reinforcing adolescents’ sense of difference from their peers who could engage with metaphorical thinking. As illustrated by Chloe’s mother below, mechanisms of treatment and expected outcomes were perceived to be obstructed by her adolescent’s unique autistic profile.

*You know they [clinicians] say “imagine that your toes are dipping into the water and how cold it is” and all that sort of thing and I can…imagine a past experience from me doing it so yes I can imagine it but [Chloe] is like “oh no they’re not so they’re not”. So most of the techniques are usually around anxiety…they’re blocked by the ASD so therefore she [Chloe] doesn’t get the benefit of a drop in anxiety. And therefore the drop in pain doesn’t come because the anxiety threshold is still there (Chloe’s mother, dyad).*

In the above example, perceived difference, particularly in relation to the processing of information given during psychological intervention, resulted in reduced benefits of this treatment for Chloe in alleviating their pain. Such perceived differences between themselves and their nonautistic peers at times resulted in the adolescents’ disapproval of, disengagement and subsequent withdrawal from psychological aspects of treatment. This served to further increase the distinction between those with chronic pain who were finding benefits in and engaging well with treatment and the autistic adolescents, who were unable to engage in managing their pain in this way.

*I was just sick of they [clinicians] were trying to like almost assign things to the pain like “what do you associate with it?” trying to avoid that basically and it was just not helping at all for me, so I ended up just leaving (Jacob, individual).*

*Anything to do with mindfulness or… any other that kind of aspect of it the psychology side of it he definitely saw it as a load of hokey and…didn’t wanna engage, didn’t wanna go near. (Jacob’s mother, individual*).

Both adolescent and maternal accounts identified that autistic adolescents had unique and differing treatment needs to their nonautistic peers, yet these were neither being understood nor met in standard treatment.

## Discussion

This study sought to understand how autistic adolescents living with chronic pain and their parents (mothers) sought to understand their experiences of pain, how pain is managed and their perceptions of psychologically led treatment for adolescent chronic pain.

Our findings suggest that autistic adolescents experience enhanced pain in the presence of heightened sensory stimulation and related anxiety. Atypical sensory processing is a common feature of autism, marked by hyper-responsiveness or hypo-responsiveness to sensory inputs (Ben-Sasson et al., 2019). Moreover, the link between altered sensory processing and anxiety in autistic young people is well documented (Johnco & Storch, 2015). While evidence supports the link between anxiety and pain in neurotypical pediatric pain settings (Fisher et al., 2018), autistic adolescents in our study described a reciprocal relationship between elevated pain and anxiety that was frequently triggered by sensory overload (Failla et al., 2020). This suggests that hyper-responsiveness to sensory input increased adolescents’ experience of anxiety and co-occurring pain. These findings are congruent with literature, which has demonstrated a strong relationship between sensory over-responsiveness and anxiety in autistic young people living with chronic gastrointestinal issues (including pain) and migraine (Mazurek et al., 2012; Sullivan et al., 2014).

To manage the dynamic relationship between sensory over-responsiveness, anxiety and pain, routine and ritualistic behaviors were adopted. Mothers highlighted how they encouraged the adoption of routine and ritualistic behaviors in response to their adolescents experiencing sensory overload and withdrawing from others. Such social withdrawal and avoidance of emotional and unpredictable cues have been observed as common responses to sensory over-responsiveness in autistic young people (Istvan et al., 2020). The adolescents in the present study did not, however, reflect upon withdrawal behaviors in the context of experiencing pain. Instead, engaging in routine and ritualistic behaviors enabled individuals to maintain a sense of control, reducing the likelihood of sensory overload and alleviating associated anxiety and pain. As the association between altered sensory processing and restricted and repetitive behaviors in autistic young people has been shown to be mediated by anxiety (Wigham et al., 2015), such ritual behaviors may develop to manage elevated anxiety that is triggered by difficulty in processing sensory inputs (Dar et al., 2012). Our study findings highlight a similar pattern within the context of chronic pain in autistic adolescents, suggesting that routine and ritualistic behaviors may develop as a strategy for coping with heightened pain in response to overstimulating sensory and anxiety-provoking events. Such behaviors may be considered beneficial in the context of pain.

Nonetheless, engaging in routine and ritualistic behaviors appeared to negatively impact autistic adolescents’ experience of pain in some ways. For example, routine and ritualistic behaviors were sometimes discussed in relation to pain medication, whereby adolescents were unable to engage flexibly with times at which medication was administered and by whom. Such behavioral invariance or rigidity is a common characteristic of autism and a specific construct of routine and ritualistic behaviors (Sethi et al., 2019). Relatedly, some mothers described their adolescents’ inflexibility around pain medication as contributing to periods of untreated pain when their nominated medication-administering parent was absent, identifying inflexibility as an unhelpful strategy for managing pain in this instance. Research involving pain-free autistic young people has shown that parents perceive such behavioral invariance to increase the levels of anxiety experienced by their child (Sethi et al., 2019). Contrastingly, our findings suggest the possible utility of routine and ritualistic behaviors for alleviating the reciprocal relationship between anxiety and pain in autistic adolescents; however, behavioral invariance may be an important construct of these behaviors that is less adaptive in the context of pain management for some autistic individuals.

Furthermore, our study findings highlighted a heightened sense of isolation associated with both being autistic and living with chronic pain, affecting adolescents’ sense of lack of fit with neurotypical peers and their experience of effective clinical pain management. Altered expressions of pain (e.g., laughing) were noted in adolescent and parental accounts in our study but were perceived as being rarely identified by clinicians as reflecting pain experience in autistic individuals. This finding supports evidence suggesting that autistic young people express physical discomfort and pain differently from neurotypical young people, leading to them being perceived as insensitive to pain or their pain being overlooked by clinicians (Allely, 2013).

### Strengths and limitations

This was the first study to qualitatively explore how autistic adolescents and their mothers perceive, understand, and experience chronic pain. Our findings may be a useful contribution to both the pain and autism literatures and to the evidence base focusing on chronic pain in neurodivergent populations. Regarding study limitations, all participants were white, all adolescent participants were older (15–19 years), the majority identified as female and all parental participants were mothers, demonstrating a lack of representation of the experiences of fathers, younger adolescents, male participants and individuals from other ethnicities. Additionally, participants engaging in this neurotypical specialist pain service likely had low support needs and as such, it is important to acknowledge that these study findings are based on analysis of accounts of autistic adolescents who were willing and able to participate in verbal interviews. Engaging adolescents from underrepresented groups (e.g., ethnicity, younger adolescents, those that identify as male, low socioeconomic status, and autistic adolescents with more substantial support needs) in future work would enhance our understanding of coexisting pain and autism for individuals who may experience additional barriers to pain management.

### Implications for research and clinical practice

**Figure 1.**
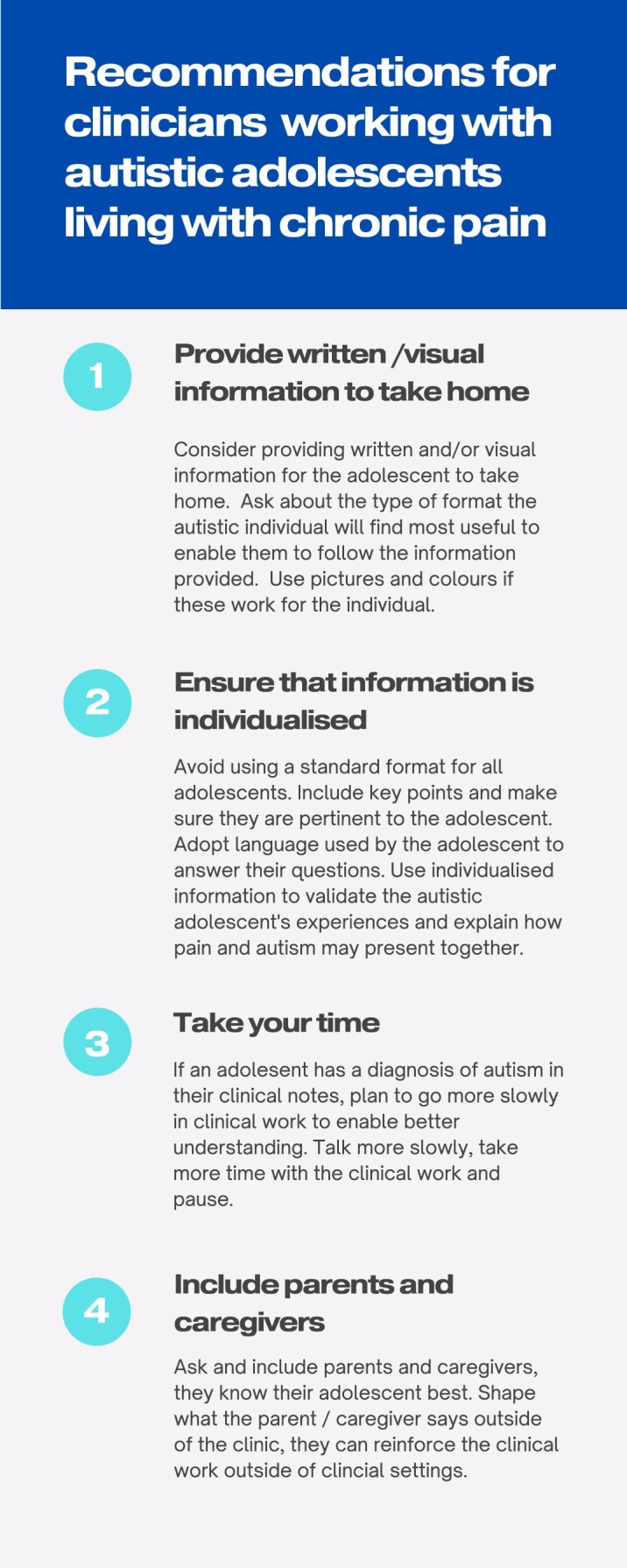
Recommendations for clinicians working with austistic adolescents living with chronic pain.

Our study findings showed that chronic pain treatment was perceived to be designed for neurotypical populations, with autistic adolescents perceiving reduced benefit from such treatment, impacting potential treatment disengagement or withdrawal. Specifically, findings showed that adolescents’ perceived difficulties with engaging with metaphorical and abstract thinking exercises that can form fundamental components of psychological interventions. This is unsurprising since evidence identifies how difficulty engaging with and understanding nonliteral language and reasoning is common in autistic individuals and thought to result from dysfunction in underlying neurolinguistic mechanisms (Gold et al., 2010). The use of metaphors is an example of nonliteral language used frequently within psychological therapies but is known to be challenging for autistic individuals who may rely on abstract concepts and connections (Kalandadze et al., 2018). Clinicians are encouraged to adapt their communication for autistic individuals to incorporate concrete language and avoid metaphors within therapy sessions (Cooper et al., 2018). Additionally, we suggest that clinicians adapt their communication style to meet the needs of autistic adolescents in terms of information format (e.g., verbal/written/pictorial), by individualizing content to meet specific individual needs and by providing such individualized information as a means to take information home from the clinical encounter to facilitate understanding. We have included further clinical recommendations in Figure 1. Increasing access to education and support for clinicians to increase their confidence and competence in assessing and treating pain in autistic populations is imperative for improving long-term pain management in autistic individuals (Liu et al., 2020) and closing the gap in treatment benefits experienced between autistic and neurotypical individuals.

While our findings suggest important changes with regard to adapting existing therapies (e.g., CBT, ACT) for autistic individuals to improve pain-related outcomes, it is important ensure that autistic individuals are at the center of any such adaptations. Previous research has suggested that modifications to psychological therapies to better meet the needs of autistic people have typically centered around the therapist’s perspective, neglecting to address the relevance and importance of lived experience in therapy design (Petty et al., 2021). Future therapeutic adaptation in the pain area must occur collaboratively with autistic adolescents and their parents.

## Conclusion

Our qualitative study findings suggest that autistic adolescents living with pain experience pain in a unique manner and face significant barriers to effective pain treatment in clinical settings. The development of bespoke educational resources for clinicians, focusing specifically on communication strategies, to better support them in working with autistic adolescents with chronic pain could helpfully improve treatment outcomes for autistic adolescents living with chronic pain.

## Supplementary Material

jsae004_Supplementary_Data

## Data Availability

Data cannot be shared for ethical/privacy reasons.
