# Supplementary material for: Understanding the impacts of chronic pain on autistic adolescents and effective pain management: a reflexive thematic analysis adolescent–maternal dyadic study
Source: J Pediatr Psychol. 2024 Feb 7;49(3):185–94. doi: 10.1093/jpepsy/jsae004 (PMC10954305; doi:10.1093/jpepsy/jsae004)
Supplement: jsae004_Supplementary_Data [file jsae004_supplementary_data.docx]

**Supplementary Materials**

Table 1: Example of the adolescent interview schedule.

| Topic area | Question | Probes and prompts |
| --- | --- | --- |
| Introduction | Can you tell me about your autism? | - When was this first identified? - How would you describe your autism? |
| Autism related -cognition | We know that some autistic people have a particular way of thinking about things. For example, they might have a very systematic way of approaching things or hold views that are hard to change. Can you tell me about your experience of this? | - How does having this particular way of thinking about things affect your ability to cope with pain and live a good life with it? - What do you find most difficult? Why? |
| Autism related – rituals and change | We know that some autistic people have a particular way of doing things. For example, they might have need to have particular routines, or to always do things in a very specific way. Can you tell me about your experiences of this? | - How does having this particular way of doing things affect your ability to cope with pain and live a good life with it? - What do you find the most difficult thing? Why? - Can you tell us about any specific interests you have in things? i.e. Are there things that you are really interested in that other people do not share? |
| Autism related – body awareness | We know that some autistic people feel especially aware of what is happening in their body. For example, they may not be aware of feeling hungry or that they want to go to the bathroom until they really need to go. Can you tell me about your experience of this? | - How does your awareness of your body affect your experience of pain and ability to cope with pain and live a good life with it? - What do you find most difficult? Why? |
| Autism related - touch | Some autistic people feel a particular way about being touched by people or things. For example, they might find being hugged difficult. How does being touched affect you? | - How do your difficulties with being touched affect your ability to cope with pain and live a good life with it? - What do you find most difficult? Why? |
| Autism related – peers and communication | Some autistic people have difficulties with friendships, or with coping with groups of people. Can you tell me about any things that you find difficult in this area? | - How do these communication difficulties affect your ability to cope with pain and have a good life with it? What do you find most difficult? Why? |
| Autism related – family and communication | Some autistic people have difficulties with communicating with people in their family. Can you tell me about any things that you find difficult in this area? | - How do these communication difficulties affect your ability to cope with pain and have a good life with it? What do you find most difficult? Why? |
| Autism related - experiencing emotional reactions) | Some people have told us that being autistic can affect their ability to feel emotions. Can you tell me about your experiences of feeling emotions? | - How do these difficulties with feeling emotions affect your ability to cope with pain and have a good life with it? |
| Autism related - displaying emotion | Some people have told us that being autistic can affect their ability to showing how they feel to others. Can you tell me about your experiences of showing how you feel to others? | - How do these difficulties with showing how you feel to others affect your ability to cope with pain and have a good life with it? - What is the most difficult thing? Why is this so difficult? - How do other people know how you are feeling? |
| Autism related - sensory sensitivities | Some autistic people can really struggle with things like loud noises, bright lights, and particular textures and feelings against their skin. Can you tell me about any difficulties you have in this area? | - How do these difficulties with noises/light/texture affect how you cope with pain and lives a good life with it? |
| Pain related - treatment | Thinking about your experience of treatment – how do you think it was affected by the fact that you are autistic? | - How do you think your treatment compares with someone who is not autistic? - What might be similar? - What might be different? |
| Pain related - treatment | How could treatment for pain be made easier for you? | - What would help? - Why would this help? |
| Pain related - treatment | We know that it often takes a long time for young people to get help for their pain. Can you tell me about how being autistic might have affected how you were able to get help for your pain. | - What has been difficult? - Why has this been difficult? |
